# A Deep CNN-LSTM Model for Particulate Matter (PM_2.5_) Forecasting in Smart Cities

**DOI:** 10.3390/s18072220

**Published:** 2018-07-10

**Authors:** Chiou-Jye Huang, Ping-Huan Kuo

**Affiliations:** 1 School of Electrical Engineering and Automation, Jiangxi University of Science and Technology, Ganzhou 341000, China; chioujye@163.com; 2 Computer and Intelligent Robot Program for Bachelor Degree, National Pingtung University, Pingtung 90004, Taiwan

**Keywords:** PM_2.5_ forecasting, deep learning, big data analytics, CNN-LSTM model

## Abstract

In modern society, air pollution is an important topic as this pollution exerts a critically bad influence on human health and the environment. Among air pollutants, Particulate Matter (PM_2.5_) consists of suspended particles with a diameter equal to or less than 2.5 μm. Sources of PM_2.5_ can be coal-fired power generation, smoke, or dusts. These suspended particles in the air can damage the respiratory and cardiovascular systems of the human body, which may further lead to other diseases such as asthma, lung cancer, or cardiovascular diseases. To monitor and estimate the PM_2.5_ concentration, Convolutional Neural Network (CNN) and Long Short-Term Memory (LSTM) are combined and applied to the PM_2.5_ forecasting system. To compare the overall performance of each algorithm, four measurement indexes, Mean Absolute Error (MAE), Root Mean Square Error (RMSE) Pearson correlation coefficient and Index of Agreement (IA) are applied to the experiments in this paper. Compared with other machine learning methods, the experimental results showed that the forecasting accuracy of the proposed CNN-LSTM model (APNet) is verified to be the highest in this paper. For the CNN-LSTM model, its feasibility and practicability to forecast the PM_2.5_ concentration are also verified in this paper. The main contribution of this paper is to develop a deep neural network model that integrates the CNN and LSTM architectures, and through historical data such as cumulated hours of rain, cumulated wind speed and PM_2.5_ concentration. In the future, this study can also be applied to the prevention and control of PM_2.5_.

## 1. Introduction

As the International Energy Agency (IEA) [1] had pointed out, air pollution causes the premature death of 6.5 million people every year [2], and thus far, energy production and utilization are the largest man-made air pollution sources. Air pollution abatement technology has become a part of public knowledge, and clean air is extremely important to ensure human health. Although people have an increasing recognition as to its urgency, air pollution problems are still unsolved in many countries, and global health risks will be extended further in future decades [2]. Among pollution sources, suspended particles with a diameter equal to or less than 2.5 μm are called PM_2.5_. As the particles of this pollution source are small, they can penetrate the alveoli, and even pass through the lungs and affects other organs of the body [3].

In some major cities of the world (e.g., New York, Los Angeles, Beijing, and Taipei), air pollution has been identified as one of the main health hazards [3]. The air pollution in big cities also negatively impacts the environment around the city. One reference [4] pointed out that high PM_2.5_ concentration has even been detected in regions such as the East China Plain, Sichuan Province, and the Taklimakan desert. Studies about the relationship between PM_2.5_ and mortality in US cities had also been discussed in detail by Kioumourtzoglou et al. [5]. Thus, for urban residents, solving PM_2.5_ air pollution is a critically urgent and important topic. Although Walsh [6] pointed out that the main PM_2.5_ pollution source for the major cities in China is motor vehicles, there are a large number of sources of air pollution, and the degree of air pollution is also related to weather and wind direction. Therefore, the management and control of city air pollution is rather complex. 

For a smart city, to create a smarter environment and improve the quality of its citizens’ lives, it is indispensable to equip the city with the functions of sensing the weather and the surrounding environment. Liu et al. [7] put forward an idea to establish a smart urban sensing system architecture using Internet of Things (IoTs) which is equipped with sensing and monitoring systems for PM_2.5_, temperature, and noise. This system can efficiently monitor the condition of air pollution and other environmental pollution of the city and collect data for analysis and strategy evaluation. Zhang et al. [8] used IoTs technology and combined information such as social media, air quality, taxi trajectory, and traffic conditions, and integrated machine learning technology, which has become one valuable application in smart cities. Zeng and Xiang [9] also proposed an air pollution sensing and monitoring system applied to smart cities. This system adopts a Q-learning algorithm to realize the computation of an adaptive sampling scheme. Apart from these studies, there are also other studies about developing various sensors to investigate air pollution. For instance, Ghaffari et al. [10] puts forward a nitrate sensor whose sensitivity and accuracy had been well verified in experiments.

Since the topic of PM_2.5_ air pollution has received increasing attention, there is currently much relevant analysis and many studies about PM_2.5_. Lary and Sattler [11] proposed a method to estimate the PM_2.5_ concentration using machine learning. This method collected the air pollution indexes from 55 countries from 1997 to 2014, and used a machine learning method for modeling. It produced decent results, but it could only carry out tiny estimation among intervals, and could not forecast future PM_2.5_ conditions. In 2016, Li et al. adopted the Stacked Autoencoder (SAE) architecture to forecast the PM_2.5_ concentration of various regions [12]. Although SAE requires the pre-training step and cannot perform training directly, its performance is good. In 2017, the latest research by Li et al. showed that the air pollution estimation system based on a Long Short-Term Memory (LSTM) neural network is more accurate [13]. Therefore, the application of LSTM to the research topic of air pollution is a good approach. Additionally, Yu et al. [14] uses an Eta-Community Multiscale Air Quality (Eta-CMAQ) forecasting model to forecast the air pollution index of PM_2.5_. This method can perform the PM_2.5_ forecasting according to the chemical composition of PM_2.5_, such as Organic Carbon (OC) or Elemental Carbon (EC). This approach belongs to the traditional PM_2.5_ forecasting algorithm, and its result is effective and feasible. However, it cannot perform comprehensive forecasting according to weather information (e.g., wind speed, rainfall, etc.).

The particles and molecules generate a light scattering phenomenon under illumination, and at the same time absorb partial energy of the illumination. When a collimated monochromatic light is projected on a measured particle field, it is affected by the light scattering and absorption around the particles, and the light intensity is attenuated. This way, the relative attenuation ratio of the light projected through the concentration field can be measured and obtained. Therefore, the relative attenuation rate can fundamentally reflect the linearity of the relative concentration of dust in the pending field. The intensity of light is proportional to the strength of the electrical signal of the optical to electrical conversion, by measuring the electrical signal, the relative attenuation rate can be obtained, and the concentration of dust in the field to be measured can be determined [15,16]. Furthermore, using geospatial assessment tools [17] or more complex statistic algorithms [18,19] are also feasible and practical for the forecasting of PM_2.5_ pollution issue.

Summing up, PM_2.5_ forecasting is absolutely a vital topic for the development of smart cities. In this paper, a deep learning model based on the main architectures of CNN and LSTM is proposed to forecast future PM_2.5_ concentration. This architecture can conduct the forecast of the future PM_2.5_ concentration according to the past PM_2.5_ concentration and even other weather conditions. To compare the overall efficiency of each algorithm, two measurement indexes, MAE and RMSE are also applied to the experiments in this paper. In addition, other traditional machine learning algorithms are compared. The performance of all algorithms is also graded and verified in each experiment. As for the aspect of database selection, a PM_2.5_ dataset of Beijing is used. Aimed at the problems in smart cities that urgently need to be solved, PM_2.5_ forecasting is integrated into the air pollution forecasting system of the smart city, thus achieving the prospect of creating a better and smarter city.

The major contributions of this paper are: (1) designing a high precision PM_2.5_ forecasting algorithm; (2) comparing the performances of the several popular machine learning methods in the air pollution forecasting problem; and (3) validating the practicality and feasibility of the proposed network in PM_2.5_ forecasting application.

This paper is organized as follows. The PM_2.5_ monitoring and forecasting in smart cities is described in Section 2; the background knowledge of the artificial neural network is presented in Section 3; the design of the proposed APNet is illustrated in Section 4; the forecasting and comparison results are demonstrated in Section 5; and conclusions are given in Section 6.

## 2. PM_2.5_ Monitoring and Forecasting in Smart Cities

**Figure 1:**
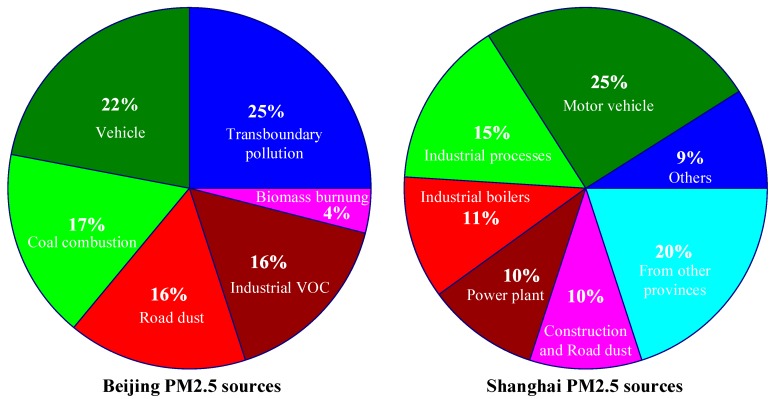
The Particulate Matter (PM)2.5 source pie charts of Beijing and Shanghai [20].

The PM_2.5_ source analyses of two major cities, Beijing and Shanghai, are shown in Figure 1 [20]. As shown, in Beijing, the biggest PM_2.5_ pollution source comes from transboundary pollution (25%), and the second biggest source is motor vehicles (22%); while in Shanghai, the biggest PM_2.5_ pollution source comes from motor vehicles (25%), and the second biggest source is pollution from other provinces (20%). This indicates that PM_2.5_ pollution caused by vehicles has a great effect on urban air pollution. As the air pollution condition can be changed to some degree by the wind direction, pollution sources from other regions is another one of the main reasons. Additionally, there are still many other factors that cause PM_2.5_ pollution, such as coal combustion, road dust, industrial Volatile Organic Compound (VOC), biomass burning, and combustion installations. All of these can affect the overall PM_2.5_ concentration of a city. Therefore, the tracking and forecasting of PM_2.5_ concentration is a challenging and important topic in smart cities. 

**Figure 2:**
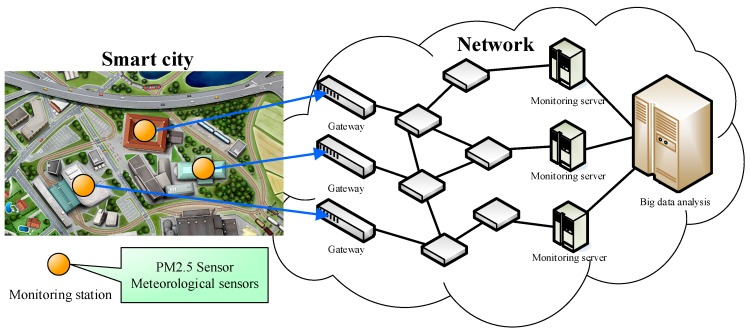
Urban sensing application in big data analysis; meteorological parameters: Temperature, Relative Humidity, and Precipitations.

To effectively monitor and forecast the PM_2.5_ concentration in smart cities, an urban sensing application in big data analysis is set up whose architecture is shown in Figure 2. First, various sensors can be installed at various corners in the city, such as PM_2.5_ sensors and meteorological sensors to sense the urban weather conditions and degree of air pollution. Next, to monitor each index effectively, Internet of Things (IoTs) can be used to transfer the information and data to the monitoring servers for performing long-term data monitoring and tracking. However, for a smart city, merely monitoring the collected data above is insufficient since the large amount of collected data are a valuable resource. Therefore, relevant big data analysis techniques can be used to analyze and track the various data so as to reach the goal of effectively monitoring, managing, and maintaining citizens’ health. In this paper, the proposed CNN-LSTM is an advanced algorithm which adopts artificial intelligence and big data, and combines various data indexes to accurately forecast the future PM_2.5_ concentration. The detailed algorithm architecture is introduced in the following sections.

## 3. The Background Knowledge of the Artificial Neural Network

An Artificial Neural Network (ANN) is a kind of mathematic model that imitates the operation of biological neuron. It is a strong, non-linear modeling tool. An earlier ANN architecture is Multilayer Perceptron (MLP) [21], a neural network with a fully-connected architecture. Basically, MLP already has a good performance, and has been applied widely. However, if the data complexity is high, the MLP architecture alone may fail to learn all the conditions effectively. At present, many new architectures have been developed for ANN. In this paper, the main architectures are Convolutional Neural Network (CNN) [22] and Long Short-Term Memory (LSTM) [23,24].

### 3.1. Convolutional Neural Network

**Figure 3:**
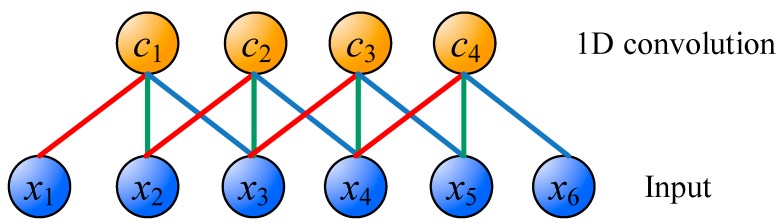
The one-dimensional (1D) convolution operation.

A one-dimensional (1D) convolution operation is shown in Figure 3. The difference between CNN and MLP is that CNN uses the concept of weight sharing. In Figure 3, *x*_1_ to *x*_6_ are inputs, and *c*_1_ to *c*_4_ are the feature maps after 1D convolution. What connects the input layer and convoluting layer are red, blue, and green connections. Each connection has its own weight value, and the connections of the same color have the same weight value. Therefore, in Figure 3, it only needs 3 weight values to perform the convolution operation. The advantage of CNN is that the training is relatively easy because the number of weights is less than that of fully-connected architecture. Moreover, important features can be effectively extracted.

### 3.2. Long Short-Term Memory

**Figure 4:**
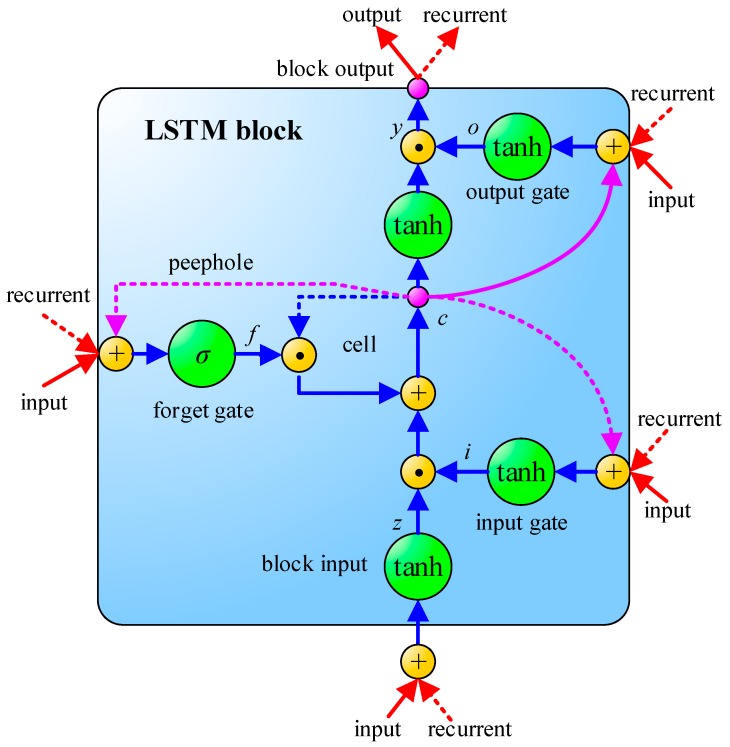
The schematic of Long Short-Term Memory (LSTM) [24].

Another important technology of ANN is Recurrent Neural Network (RNN), which differs from CNN and MLP in its consideration of the time sequence. LSTM [18] is one of the RNN models. The schematic of LSTM is shown in Figure 4, where σ is a sigmoid function, as shown in Equation (1). LSTM contains an input gate, an output gate and a forget gate. The interactive operation among these three gates makes LSTM have the sufficient ability to solve the problem of long-term dependencies which general RNNs cannot learn. In addition, a common problem in deep neural networks is called gradient vanishing, i.e., The learning speed of the previous hidden layers is slower than the deeper hidden layers. This phenomenon may even lead to a decrease of accuracy rate as hidden layers increase [25]. However, the smart design of the memory cell in LSTM can effectively solve the problem of gradient vanishing in backpropagation and can learn the input sequence with longer time steps. Hence, LSTM is commonly used for solving applications related to time serial issues. The specific formula derivation of LSTM is illustrated in Equations (2)–(11):

(1) $$\mathrm{sigmoid}(x)=\frac{1}{1+e^{-x}}$$

(2) $${\bar{z}}^{t}=W_{z}x^{t}+R_{z}y^{t-1}+b_{z}$$

(3) $$z^{t}=\tanh\left({\bar{z}}^{t}\right)$$

(4) $${\bar{i}}^{t}=W_{i}x^{t}+R_{i}y^{t-1}+p_{i}⊙c^{t-1}+b_{i}$$

(5) $$i^{t}=\mathrm{sigmoid}\left({\bar{i}}^{t}\right)$$

(6) $${\bar{f}}^{t}=W_{f}x^{t}+R_{f}y^{t-1}+p_{f}⊙c^{t-1}+b_{f}$$

(7) $$f^{t}=\mathrm{sigmoid}\left({\bar{f}}^{t}\right)$$

(8) $$c^{t}=z^{t}⊙i^{t}+c^{t-1}⊙f^{t}$$

(9) $${\bar{o}}^{t}=W_{o}x^{t}+R_{o}y^{t-1}+p_{o}⊙c^{t-1}+b_{o}$$

(10) $$o^{t}=\mathrm{sigmoid}\left({\bar{o}}^{t}\right)$$

(11) $$y^{t}=\tanh\left(c^{t}\right)⊙o^{t}$$

where *W_z_*, *W_i_*, *W_f_*, and *Wo* are input weights; *R_z_*, *R_i_*, *R_f_*, and *R_o_* are recurrent weights, *p_i_*, *p_f_*, and *p_o_* are peephole weights; *b_z_*, *b_i_*, *b_f_*, and *b_o_* are bias weights; *z^t^* is the block input gate; *f^t^* is the forget gate; *c^t^* is the cell; *o^t^* is the output gate; *y^t^* is the block output; and ⊙ represents point-wise multiplication. To reach the goal of parameter optimization, either CNN or LSTM can use backpropagation to adjust the parameters of the model during the process of training.

### 3.3. Batch Normalization

During the training of deep neural network, some problems still emerge. For instance, due to the large number of layers within deep neural networks, a change of the parameters of one layer can usually affect the outputs of all the succeeding layers, which leads to frequent parameter modifications, and thus, a low training efficiency. Additionally, before passing the activation function, if the output value of a nerve cell exceeds dramatically the appropriate range of the activation function itself, it may also result in the failure of the work of the nerve cell. To solve these problems, batch normalization [26] is designed. The detailed formulas of batch normalization are shown in Equations (12)–(15):

(12) $$\mu_{B}=\frac{1}{m}\sum_{i=1}^{m}x_{i}$$

(13) $$\sigma_{B}^{2}=\frac{1}{m}\sum_{i=1}^{m}{\left(x_{i}-\mu_{B}\right)}^{2}$$

(14) $${\hat{x}}_{i}=\frac{x_{i}-\mu_{B}}{\sqrt{\sigma_{B}^{2}+\varepsilon }}$$

(15) $$y_{i}=\gamma {\hat{x}}_{i}+\beta \equiv BN_{\gamma ,\beta }\left(x_{i}\right)$$

where *x_i_* is the input value and *y_i_* is the output after batch normalization; *m* refers to the mini-batch size, i.e., the one mini-batch that has m inputs; $\mu_{B}$ is the mean of all the inputs in the same mini-batch; and $\sigma_{B}^{2}$ is the variance of the input in a mini-batch. Next, according to the values of $\mu_{B}$ and $\sigma_{B}^{2}$, all the *x_i_* are normalized as ${\hat{x}}_{i}$ and substituted into Equation (15) to obtain *y_i_*, in which *γ* and *β* are learnable parameters. Through batch normalization, the neurons in the deep neural network can be fully exploited and the training efficiency can be improved.

## 4. The Proposed Deep CNN-LSTM Network

**Figure 5:**
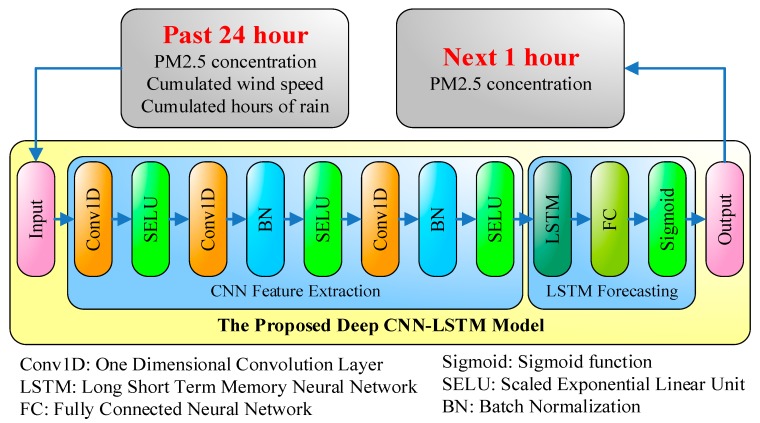
The architecture of the proposed APNet.

The architecture of the proposed APNet is shown in Figure 5. The inputs of APNet are the records of the PM_2.5_ concentration, cumulated wind speeds, and cumulated hours of rain over the last 24 h. The output is the PM_2.5_ concentration of the next hour. Different from traditional pure CNN or pure LSTM architectures, the first half of APNet is CNN, and used for feature extraction. The latter half of APNet is LSTM forecasting, which is used to analyze the features extracted by CNN and then to estimate the PM_2.5_ concentration of the next point in time. The CNN part of the APNet contains three 1D convolution layers. Moreover, to improve the efficiency, batch normalization is added after the second and third convolution layers of the APNet. 

Usually Rectified Linear Unit (ReLU), as shown in (6), is widely used as the activation function. However, for the activation function of APNet here, Scaled Exponential Linear Units (SELU), as shown in (7), is used. This is because, compared with ReLU, SELU has better convergence and can effectively avoid the problem of gradient vanishing, which is discussed specifically in Klambauer et al. [27]. In Equation (7), *λ* = 1.05, *α* = 1.67, and the numerical values are specifically defined by Klambauer et al. [27]. The output of LSTM goes through the fully-connected architecture and the sigmoid activation function to produce the final output. The results represent the PM_2.5_ concentration of the next point in time.

(16) ReLU(x)=max(0,x)

(17) $$\mathrm{SE}\mathrm{LU}(x)=\lambda \left\{ \begin{array}{ll} x & \mathrm{if}\;x>0\\ \alpha e^{x}-\alpha & \mathrm{otherwise} \end{array}\right.$$

**Figure 6:**
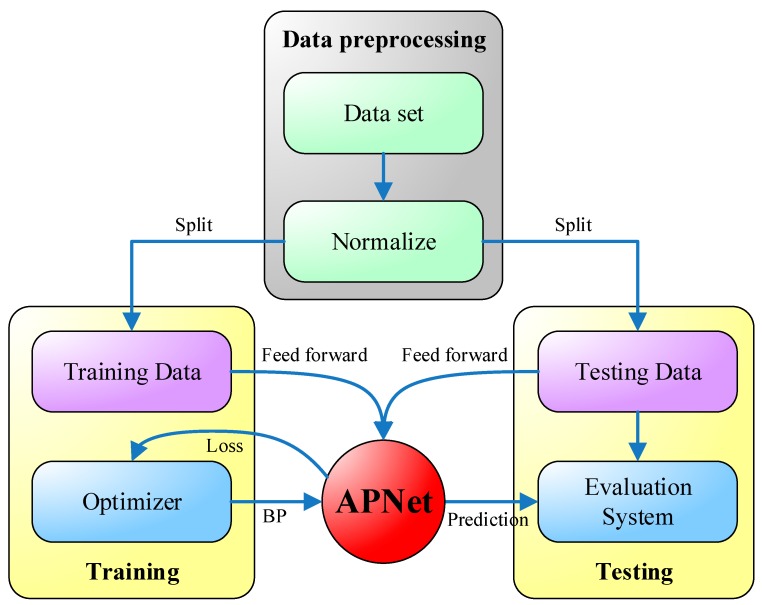
The system flow diagram of the proposed APNet.

The system flow diagram of the proposed APNet is shown in Figure 6. During data processing, the original dataset first normalized, i.e., the numerical values of all dimensions are restricted to a range of 0 to 1, so as not to be overly partial to a certain dimension during training. Next, the normalized data is separated into two parts: training data and testing data. To keep the impartiality of performance evaluation, only the training data is used during the training, while the testing data is not used. Each time the training data are input to the APNet, a loss value is generated, according to which the optimizer uses a backpropagation method to adjust the parameters of APNet. The forecast result of APNet will be more and more accurate with the increase of training iterations. After the APNet training is finished, the testing data is input into the APNet, and the testing results and real results are compared to evaluate the performance of the APNet.

When there is not enough training data or when there is overtraining, overfitting may occur. However, there are many ways to avoid overfitting, such as regularization [28], data augmentation [22], dropout [29], dropconnect [30], or early stopping [31]. Regularization, which is very popular in the field of deep learning, can be divided into L1 regularization and L2 regularization. Both of these methods will reduce the weight value of the neuronal network as much as possible to prevent overfitting [32]. The concept of data augmentation is to amplify the dataset as much as possible, for example adding random bias or noise, etc., to make the training data more diversified to achieve better training results. Dropout is similar to the dropconnect concept in that the former randomly stops the operation of the neuro, while the latter removes the connection randomly. The method used in this paper is early stopping. Before the experiment, we decided when to stop training according to the prediction condition of the validation data. For example, when training loss continues to decrease but validation loss increases, this means there is already overfitting [31], so at this time we would stop training. In the experiment, we selected an epoch value that does not generate overfitting, and let each neural network model be trained based on this epoch to maintain the fairness of the performance comparison.

## 5. Experimental Results and Discussion

This section is divided into two parts: data descriptions and experimental results. Support Vector Machine (SVM) [33,34,35,36,37,38], Random Forest (RF) [39,40,41,42,43,44], Decision Tree (DT) [45,46,47,48,49,50], MLP, CNN, and LSTM are used for comparison to fully demonstrate the performance of the proposed APNet.

### 5.1. Data Descriptions

**Figure 7:**
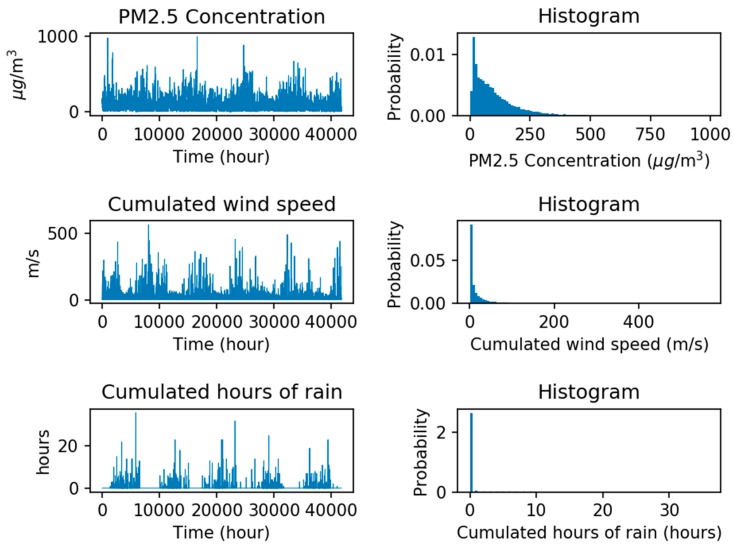
Beijing PM_2.5_ dataset.

Beijing is a cosmopolis with a population of more than 21.5 million, and Particulate Matter (PM) is one of the main factors that affect human health directly [51]. Thus, the PM_2.5_ dataset of Beijing is selected for this study. Figure 7 shows the weather condition, pollution degree reported and its histograms in each hour by the US embassy in Beijing, China, from 2010 to 2014. The dataset includes PM_2.5_ concentration, cumulated wind speed, and cumulated hours of rain. In this experiment, information from these factors over the past 24 h are used to forecast the PM_2.5_ concentration of the next hour. These three types of useful information are expected to be integrated into the machine learning model to perform supervised learning and analysis, to realize accurate forecasting.

### 5.2. Experiment Results

**Figure 8:**
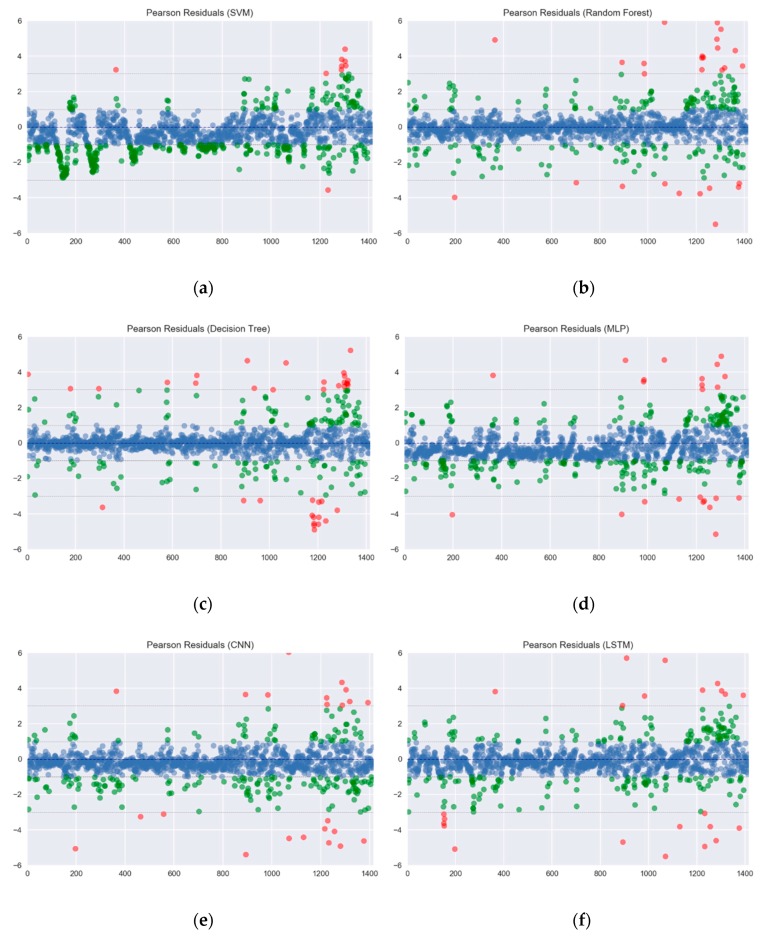

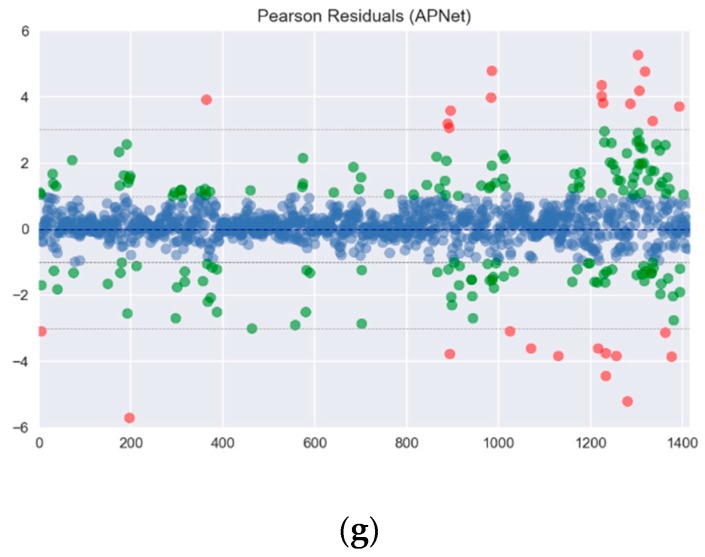
The Pearson residuals of all forecasting methods: (**a**) Partial results A; (**b**) Partial results B; (**c**) Partial results C; (**d**) Partial results D; (**e**) Partial results E; (**f**) Partial results F; (**g**) Partial results G.

In this experiment, Mean Absolute Error (MAE), Root Mean Square Error (RMSE), Pearson correlation coefficient and Index of Agreement (IA) are taken for the performance evaluation. These four kinds of measurement indexes with their equations are shown in (18)–(21). *r* is the Pearson correlation coefficient. *p_n_* denotes the predicted value, and *o_n_* represents the observed values. $\bar{o}$ is the average value of *o_n_*, and *N* is the predicted length. To test the performance comprehensively, 10 intervals in the database are selected, with each interval containing six months’ data as training data, and two months’ data as testing data. The Pearson residuals of all forecasting methods is shown in Figure 8. The results are distinguished between those with an absolute value less than 1, an absolute value between 1 and 3, and an absolute value greater than 3, the results are plotted as shown in Figure 8. From the statistical results, it can be found that the distribution of the Pearson residuals for each machine learning is not too wide, this also means that these methods have a considerable degree of predictability.

(18) $$\mathrm{MAE}=\frac{1}{N}\sum_{n=1}^{N}\left|o_{n}-p_{n}\right|$$

(19) $$\mathrm{RMSE}=\sqrt{\frac{\sum_{n=1}^{N}{\left(o_{n}-p_{n}\right)}^{2}}{N}}$$

(20) $$r=\frac{N\sum_{n=1}^{N}o_{n}p_{n}-\sum_{n=1}^{N}o_{n}\sum_{n=1}^{N}p_{n}}{\sqrt{N\sum_{n=1}^{N}o_{n}^{2}-{\left(\sum_{n=1}^{N}o_{n}\right)}^{2}}\sqrt{N\sum_{n=1}^{N}p_{n}^{2}-{\left(\sum_{n=1}^{N}p_{n}\right)}^{2}}}$$

(21) $$\mathrm{IA}=1-\frac{\sum_{n=1}^{N}{\left(\left|p_{n}-o_{n}\right|\right)}^{2}}{\sum_{n=1}^{N}{\left(\left|p_{n}-\bar{o}\right|+\left|o_{n}-\bar{o}\right|\right)}^{2}}$$

**Figure A1:**
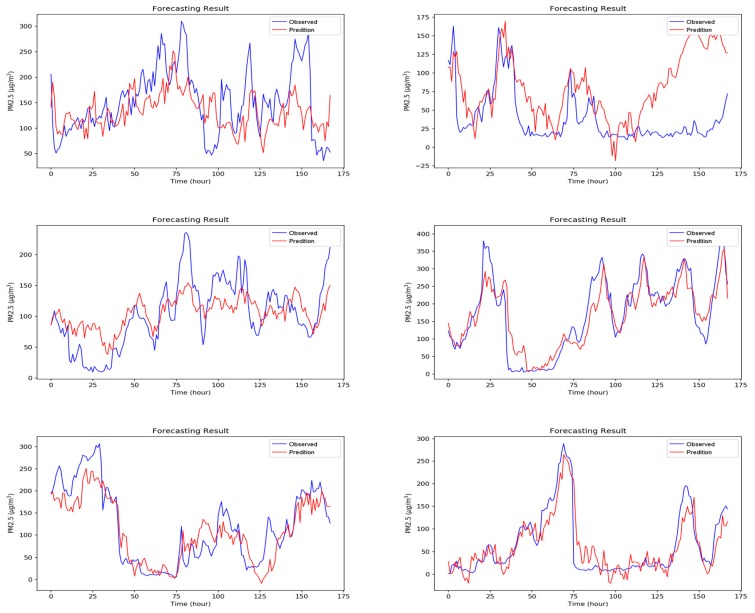
The forecasting results of Support Vector Machine (SVM).

**Figure A2:**
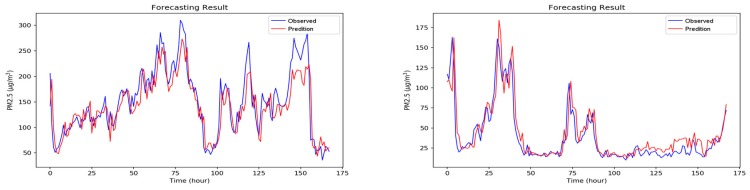

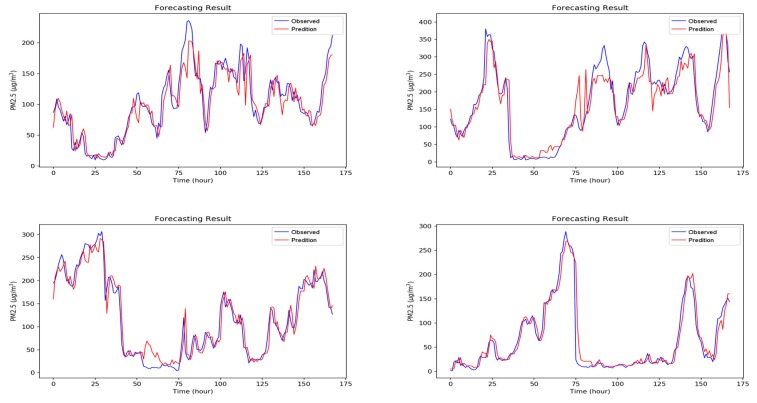
The forecasting results of random forest.

**Figure A3:**
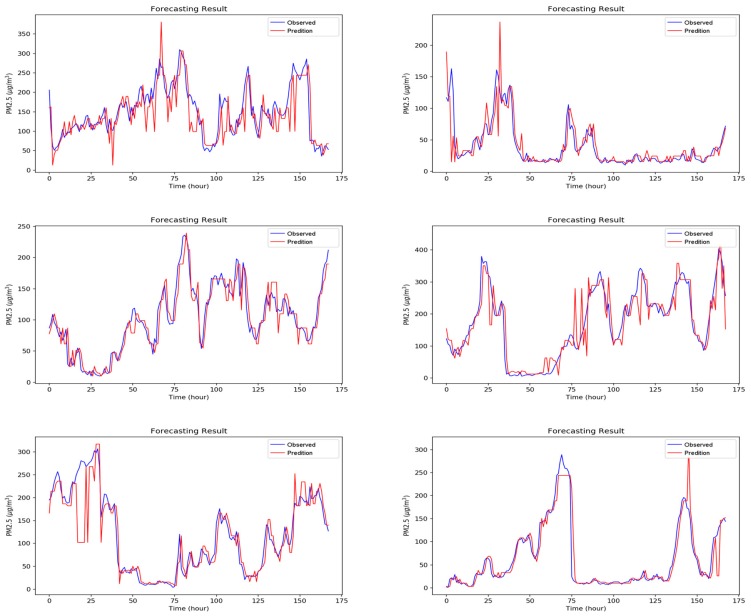
The forecasting results of decision tree.

**Figure A4:**
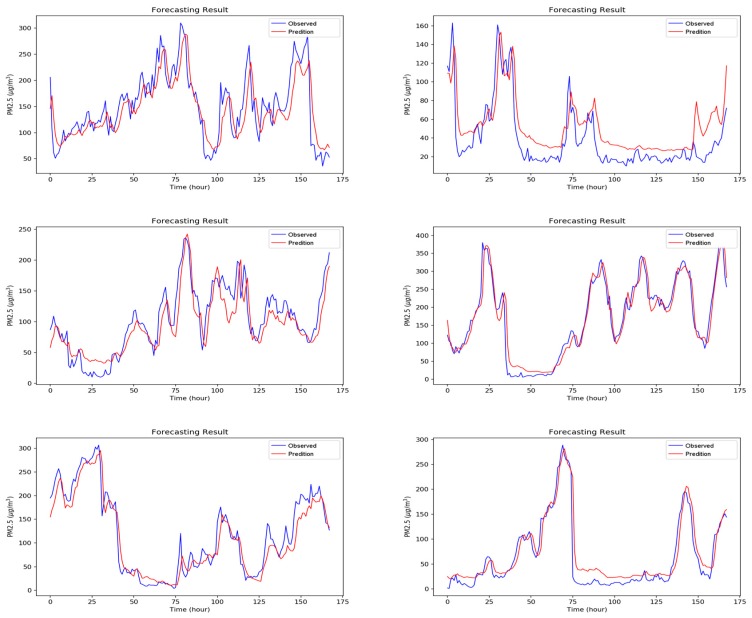
The forecasting results of Multilayer Perceptron (MLP).

**Figure A5:**
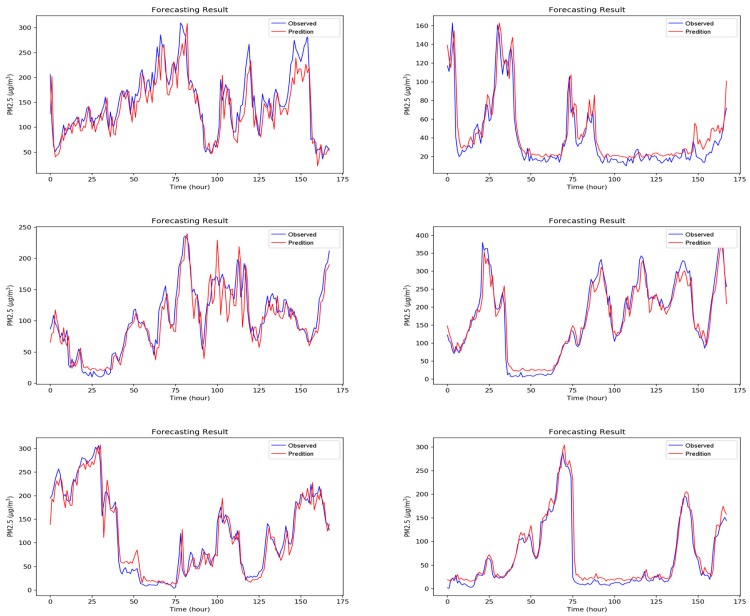
The forecasting results of Convolutional Neural Network (CNN).

**Figure A6:**
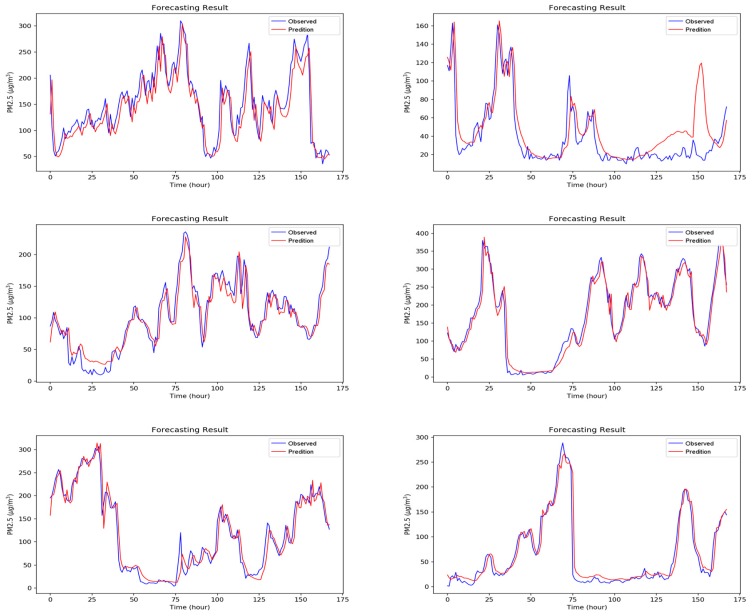
The forecasting results of LSTM.

**Figure A7:**
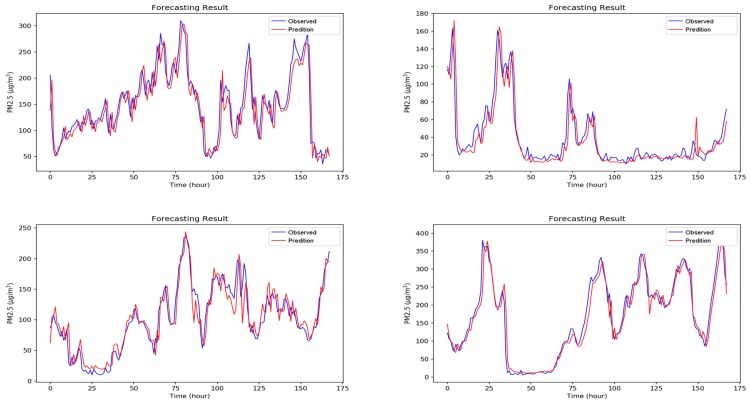

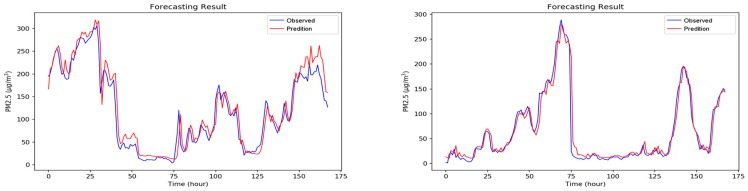
The forecasting results of the proposed APNet.

**Figure A8:**
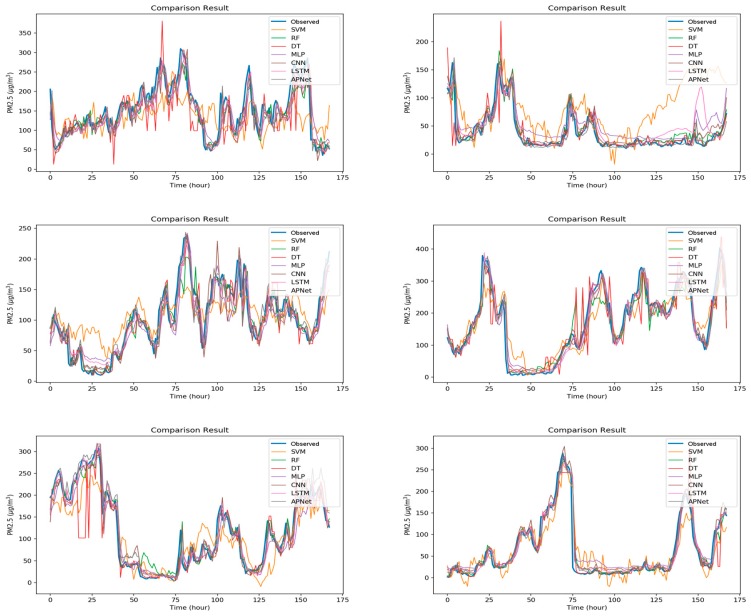
The comparisons of all the forecasting results.

Figure A1, Figure A2, Figure A3, Figure A4, Figure A5, Figure A6 and Figure A7 in Appendix A are the forecast results from each algorithm, and Figure A8 is the forecast results comparison of all the algorithms. In order to be able to perform a more complete evaluation of the effectiveness of all algorithms, we devised 10 tests for the experiments of this paper. Considering the length of this paper, we only list the results of six tests in Figure A1, Figure A2, Figure A3, Figure A4, Figure A5, Figure A6, Figure A7 and Figure A8, the detailed numerical analysis and comparison is presented in detail in Table 1, Table 2, Table 3 and Table 4. From the figures, it can be found that SVM is slightly weak on PM_2.5_ forecasting and deviated greatly from the trend of the real result at some parts. Although the performance of DT is a little better than SVM, its error is still large. The efficiencies of MLP and RF are acceptable. Although at some parts the forecasting is still not accurate, the overall trend followed that of the real results. It should be noted that the efficiency of the CNN-LSTM based APNet proposed in this paper is better than that of CNN and LSTM. Therefore, it is proven that the application of APNet to PM_2.5_ forecasting is quite effective and accurate. In these experiments, the computer specifications used for the experiment of this paper are described below: CPU: Intel Xeon E3-1245 V6; Random Access Memory (RAM): 8 GB DDR4; graphics card: GTX 1080 Ti; hard disk drive: 1 TB SATA3; Operating System: Linux Ubuntu 16.04. Because the calculation times for predicting PM_2.5_ concentration through various algorithms are all very short, all experiment methods used in this paper are within a reasonable range for predicting PM_2.5_ concentration for the next hour.

Moreover, the detailed MAE, RMSE, Pearson correlation coefficient, and IA values are shown in Table 1, Table 2, Table 3 and Table 4. In the ranking of MAE, there are, from low to high, APNet (14.63446), LSTM (15.34655), CNN (16.13498), RF (17.56451), MLP (20.65027), DT (21.41757), and SVM (37.90581). While in the ranking of RMSE, there are, from low to high, APNet (24.22874), LSTM (24.2925), CNN (24.59636), RF (28.87602), MLP (29.09238), DT (39.45547), and SVM (50.02137). Besides, in the ranking of Pearson correlation coefficient, there are, from high to low, APNet (0.959986), CNN (0.958363), LSTM (0.95794), RF (0.942276), MLP (0.940557), DT (0.880291), and SVM (0.81133). Finally, in the ranking of IA, there are, from high to low, APNet (0.97831), LSTM (0.976797), CNN (0.975972), RF (0.966298), MLP (0.964021), DT (0.933533), and SVM (0.852398). Experiments show that the APNet algorithm proposed in this paper is very good when the Pearson correlation coefficient is presented, in which the first, third, fifth, seventh, eighth, and tenth tests all have the highest r value, and the average value is also the best among all machine learning methods. In terms of IA, APNet also scored highest in IA in the first, third, fifth, seventh, eighth, and tenth tests, the average score is also the best. Overall, CNN, LSTM, and APNet are the best performers; while APNet, which combines the advantages of CNN and LSTM, wins out. This result also confirms that the combination of CNN and LSTM is very effective for the prediction of PM_2.5_. As shown by the experiment results, the performances of CNN and LSTM are both good, but that of APNet is even better. It is also proven that for PM_2.5_ air pollution source forecasting, it is very beneficial to first perform feature extraction using CNN, and then input the feature values into the LSTM architecture.

**Figure 9:**
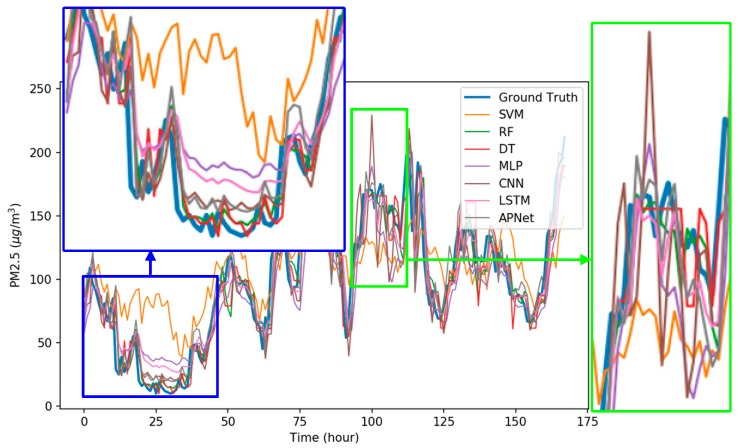
The comparisons details of forecasting results.

Figure 9 shows the detailed comparison results of each model, where the blue bold line refers to the real data, and the other colored lines are the forecast results of each algorithm. As shown in the blue frame of Figure 9, the forecast results of SVM barely coincided with the actual results. Among all the algorithms, the performances of RF, MLP, CNN, LSTM, and APNet are better. As shown in the green frame of Figure 9, when the PM_2.5_ pollution source concentration is unstable, the forecasting result of many algorithms could not follow the real trend and showed a rather disordered pattern. This also indicates that it is still difficult in terms of PM_2.5_ forecasting. Overall, the performances of CNN and LSTM are very stable and accurate, but the CNN-LSTM based APNet proposed in this paper is even better. The forecasting ability of APNet for PM_2.5_ forecasting is also verified in this experiment.

For ease of analysis, we classified air quality according to PM_2.5_ concentration as follows: Good: PM_2.5_ does not exceed 35 μg/m^3^; Pollution: PM_2.5_ is greater than 35 μg/m^3^; Severe Pollution: PM_2.5_ is greater than 150 μg/m^3^. Good quality air conditions appear in Beijing for about 23% of the time, more than half of the time (about 55%), the city is in a state of general pollution; about 22% of the time Beijing is in a state of serious pollution, general pollution and severe pollution together accounts for 77%. The proportion of the three air quality conditions has not changed much from 2010 to 2014. Compared to spring and summer, more days of clean air and severe pollution exist during autumn and winter. The former is due to Beijing’s northerly winds in autumn and winter, which facilitates air diffusion and increases the proportion of clean air. The latter is likely due to winter heating and straw burning during autumn, which causes heavy pollution to occur frequently, so the proportion of serious pollution is also relatively high. The proportion of severe pollution days in summer in Beijing is less than 17%, but the proportion of clean air days in the summer is also the lowest among the four seasons with less than 16%. Although emission from residential heating using coal is lower in summer than in winter, the temperature and humidity is higher in Beijing in the summer; at the same time, the northerly winds are reduced in summer and wind speed is low, some factors are favorable for the generation of secondary aerosols and PM_2.5_ concentration increases [52].

Because the concentration of PM_2.5_ is closely related to city area, urban population, number of vehicles, and urban industrial activity increase [53], this paper proposes a prediction model (APNet) to make short term predictions of PM_2.5_ concentrations in order to provide more effective and accurate early warnings of high concentrations of suspended particulate matter, in order to protect the people’s respiratory health and prevent cardiovascular disease.

The advantages of separate monitoring are as follows: (1) From an academic research point of view, the shorter the monitoring data collection cycle the better, that is, the more data collected in the same time period, the more applicable research can be done in the future, because the data sampling period required for each applied research is different, so separate monitoring can avoid the failing of missing data; (2) Before smart city is reached, there are still many researches and technological developments that need big data to support. In the future, big data will become a very important research asset. Figure 2 is only a schematic diagram, it is not necessary to measure data at different locations during the data collection process, it could also be done at the same location. However, in the smart city, sensors could be installed more densely in different locations so that the smart city and even neighboring areas are covered with a network of sensors, and more innovative prediction algorithms can be developed and more accurate spatiotemporal data analysis can be achieved.

The main contribution of this paper is to develop a deep neural network model that integrates the CNN and LSTM architectures, and through historical data such as cumulated hours of rain, cumulated wind speed and PM_2.5_ concentration. We allow this model to use such information to learn and predict PM_2.5_ concentration for the next hour. In the experiment process, the testing data is entirely new for the neural network model, the purpose being to verify the predictive power of APNet developed in this paper. The APNet predicted results are also analyzed and compared based on actual observed values to verify the performance of each forecasting model. Therefore, in addition to modeling past data, APNet’s output value also represents the forecasting result.

This paper mainly applies the deep neural network method to predict PM_2.5_, and compares it with many other popular and widely used machine learning algorithms. However, deep neural network is also a type of machine learning, whether the data is sufficient and correct will determine the success or failure of the algorithm prediction. Therefore, when using machine learning for data molding or forecasting, data collection and processing is very important. This does not mean however that the traditional rule-based approach is superior, because in modern society with large data resources, machine learning technology can more subtlety discover information that humans cannot intuitively reflect, and thus produce more accurate forecasts.

## 6. Conclusions

In this paper, a deep neural network model (APNet) based on CNN-LSTM is proposed to estimate PM_2.5_ concentration. APNet can forecast the PM_2.5_ concentration of the next hour according to the PM_2.5_ concentration, cumulated wind speed, and cumulated hours of rain over the last 24 h. A PM_2.5_ dataset of Beijing was used in this experiment to perform model training and performance evaluation. The experimental data in this paper were classified into two parts: training data and testing data. Training data was used for model training. The testing data that was unused in the training process was used for the computation of MAE, RMSE, Pearson correlation coefficient, and IA for performance evaluation, the results of which were comprehensively compared with that of the SVM, RD, DT, MLP, CNN, and LSTM architectures. Experimental results showed that compared with the traditional machine learning methods, the forecasting performance of the APNet proposed in this paper was proven to be the best, and its average MAE and RMSE were both the lowest. As for the CNN-LSTM based model, its feasibility and practicality for forecasting the PM_2.5_ concentration were also verified in this paper. This technology is significantly beneficial for improving the ability of estimating the air pollution in smart cites. In the future, this study can be applied to the prevention and control of PM_2.5_. In particular, in light of the severe situation of atmospheric particulate matter pollution in recent years, we must come up with appropriate countermeasures to curb the deterioration of urban air conditions. However, an urban forest can be introduced as a large air filter which is non-toxic, harmless, and non-polluting, and also saves time, labor, and resources in reducing air pollution. Urban forests have the effect of preventing air particles from lingering in the air, and it also controls and eliminates airborne particles. Research in this area may become a new direction for regulating airborne particulates with plants [54].

## Figures and Tables

**Table 1:** The experimental results in terms of Mean Absolute Error (MAE).

Test	SVM	RF	DT	MLP	CNN	LSTM	APNet
**#1**	42.57556	18.68328	23.90568	22.4221	18.9675	18.5217	16.7474
**#2**	35.40574	14.92391	19.53063	22.0437	14.8997	16.2908	14.2053
**#3**	43.37174	16.74816	17.93104	20.2441	16.9613	15.8297	14.9131
**#4**	50.19538	31.64949	36.57292	23.1328	20.7791	18.1417	18.2807
**#5**	40.38873	19.54953	27.66294	22.8951	17.1051	16.505	17.2492
**#6**	34.57838	17.80561	21.3065	18.5993	15.1543	13.9768	14.0047
**#7**	37.10853	12.3846	15.37398	19.9247	15.3203	13.1789	11.9718
**#8**	21.85433	9.96139	11.07522	13.9672	11.1243	11.1574	9.85554
**#9**	40.47121	21.13339	25.09194	26.0607	18.954	17.2029	18.9953
**#10**	33.1085	12.80574	15.72481	17.213	12.0842	12.6606	10.1216
**Average**	37.90581	17.56451	21.41757	20.65027	16.13498	15.34655	14.63446

**Table 2:** The experimental results in terms of Root Mean Square Error (RMSE).

Test	SVM	RF	DT	MLP	CNN	LSTM	APNet
**#1**	56.55255	26.59535	36.90484	29.98992	26.36855	25.2699	23.83181
**#2**	47.07641	26.84212	38.17991	30.86026	25.24918	27.20435	25.95273
**#3**	55.9933	25.46634	29.14463	27.68189	24.43146	23.31643	22.56656
**#4**	66.58581	47.20812	58.96869	35.14076	31.38514	29.63356	31.08485
**#5**	50.32762	31.14631	55.65785	31.59871	26.4418	27.15832	26.77069
**#6**	47.23936	32.32307	43.69507	27.00565	23.87708	23.05538	24.81823
**#7**	48.11796	22.96514	33.33885	28.78185	24.29253	23.04227	20.83558
**#8**	27.70533	16.61144	19.44406	19.52802	16.63667	17.22178	16.44391
**#9**	57.49434	39.29988	44.9455	38.8347	31.03137	30.14096	35.23974
**#10**	43.12105	20.30241	34.27529	21.50208	16.24985	16.88207	14.7433
**Average**	50.02137	28.87602	39.45547	29.09238	24.59636	24.2925	24.22874

**Table 3:** The Pearson correlation coefficient (*n* = 1415).

Test	SVM	RF	DT	MLP	CNN	LSTM	APNet
**#1**	0.638786	0.926131	0.857044	0.907166	0.935633	0.940295	0.941237
**#2**	0.92699	0.973356	0.945972	0.968823	0.977848	0.973044	0.975517
**#3**	0.754792	0.944363	0.926856	0.936873	0.950255	0.953075	0.955411
**#4**	0.872546	0.924315	0.868861	0.957647	0.970539	0.970023	0.966768
**#5**	0.70376	0.893368	0.699291	0.89043	0.922092	0.919221	0.932416
**#6**	0.870895	0.938605	0.879954	0.956404	0.966881	0.967185	0.964074
**#7**	0.843806	0.966459	0.927678	0.947582	0.964757	0.966151	0.972383
**#8**	0.887029	0.957205	0.943408	0.941748	0.95875	0.953544	0.96088
**#9**	0.914454	0.959145	0.940049	0.961928	0.9731	0.97354	0.963773
**#10**	0.700245	0.939808	0.8138	0.936971	0.963777	0.963319	0.967397
**Average**	0.81133	0.942276	0.880291	0.940557	0.958363	0.95794	0.959986

**Table 4:** The Index of Agreement (IA).

Test	SVM	RF	DT	MLP	CNN	LSTM	APNet
**#1**	0.745175	0.958607	0.923722	0.943082	0.959601	0.963882	0.968546
**#2**	0.952324	0.98613	0.972305	0.980782	0.988124	0.985715	0.987253
**#3**	0.716799	0.968534	0.962342	0.964832	0.972961	0.974219	0.976896
**#4**	0.873168	0.95108	0.92713	0.975282	0.979759	0.983128	0.981386
**#5**	0.790755	0.940903	0.82489	0.93198	0.958817	0.957693	0.961527
**#6**	0.897091	0.960562	0.924618	0.974253	0.982193	0.982024	0.978416
**#7**	0.904886	0.982324	0.961747	0.970803	0.979047	0.982588	0.985856
**#8**	0.924705	0.977994	0.97085	0.967449	0.977596	0.975862	0.979732
**#9**	0.934477	0.973919	0.967458	0.974426	0.984648	0.985924	0.980962
**#10**	0.784602	0.962931	0.900264	0.957321	0.976973	0.976935	0.982527
**Average**	0.852398	0.966298	0.933533	0.964021	0.975972	0.976797	0.97831
